# Cupric oxide inclusions in cuprous oxide crystals grown by the floating zone method

**DOI:** 10.1088/1468-6996/16/3/034901

**Published:** 2015-05-08

**Authors:** Laszlo Frazer, Kelvin B Chang, Kenneth R Poeppelmeier, John B Ketterson

**Affiliations:** 1 Department of Chemistry, Temple University, 1901 N. 13th Street, Philadelphia, PA 19122, USA; 2 Department of Chemistry, Northwestern University, 2145 Sheridan Road, Evanston, IL 60208-3112, USA; 3 Chemical Sciences and Engineering Division, Argonne National Laboratory, 9700 South Cass Avenue, Argonne, IL 60439, USA; 4 Department of Electrical Engineering and Computer Science, Northwestern University, 2145 Sheridan Road, Evanston, IL 60208-3112, USA

**Keywords:** cuprous oxide, inclusions, floating zone, oxides

## Abstract

Phase-pure cuprous oxide (Cu_2_O) crystals are difficult to grow since cupric oxide can form within the crystal as the crystal is cooled to ambient conditions. Vacancies are the solute which causes precipitation of macroscopic defects. Therefore, even when a mostly phase-pure single crystal is used as a feed rod, cupric oxide inclusions persist in the recrystallized solid. Control of the thermal profile during crystal growth, however, can improve phase-purity; a slow counter-rotation rate of the feed and seed rods results in fewer inclusions. Cupric oxide can be removed by annealing, which produces a factor of 540 ± 70 increase in phase-purity.

## 1. Introduction

High quality single crystals of cuprous oxide are required for optical experiments such as third harmonic generation [1, 2] and exciton scattering [3–5]. Cuprous oxide also has potential as a photovoltaic and photocatalytic material since it is composed of non-toxic, Earth-abundant elements and has a high theoretical solar energy efficiency [6–11]. While single crystals can be found in nature as the mineral cuprite, natural samples are difficult to obtain and can vary in purity and quality [12, 13]. Synthetic routes [14–18] to grow cuprous oxide crystals are therefore needed to rapidly obtain samples whose purity and quality can be systematically determined. Though small single crystals can be grown from the thermal oxidation of copper metal [19, 20] or from solution using hydrothermal methods [18, 21], crystals grown from a melt have yielded the largest and most thoroughly characterized crystals.

Molten cuprous oxide will react with many crucible materials, which makes growth from a melt difficult with techniques that require use of a crucible. Cuprous oxide can be grown using the Czochralski method with a magnesia crucible, although some reactivity is still observed [22]. We have used the crucible-free floating zone method to prevent contamination during cuprous oxide crystal growth [23–28].

Three prominent defects are present in these crystals. On the atomic scale, copper vacancies are the dominant defect [29]. On the macroscopic scale, some gas inclusions or voids can be found in cuprous oxide crystals [19, 20, 23, 30]. Cupric oxide (CuO) inclusions are also present since cupric oxide is the thermodynamic phase at ambient conditions [26]. In this paper we manipulate the macroscopic inclusions in cuprous oxide single crystals.

While a surface layer of cupric oxide can be expected if the crystal is grown in the presence of oxygen, it is perhaps initially surprising to find that cupric oxide inclusions are also found within the bulk of the crystal since the oxidation of cuprous oxide occurs primarily through the outward migration of copper atoms (as opposed to the inward migration of oxygen atoms) [31–34]. Furthermore, the presence of cupric oxide inclusions persists in crystals grown under reduced oxygen pressures [25–27].

In this study, we find inclusions also exist when crystals are grown under argon, even though the surface cupric oxide layer does not form. This suggests that the oxygen in the atmosphere does not directly influence cupric oxide inclusion formation and there is another mechanism that occurs within the bulk of the crystal.

Conversion between copper vacancies in cuprous oxide and the cupric oxide phase allows cupric oxide inclusions to form within the bulk of the crystal even in an oxygen-free atmosphere [23, 26]. While cupric oxide inclusions can ultimately be removed in thin slices through annealing procedures, bulk single crystals larger than 1mm thick that do not contain macroscopic defects are still difficult to obtain. Inclusions are opaque and can induce strain [35, 36] in bulk crystals; they may be undesirable based on the intended applications.

This work examines the effect of crystal growth conditions on macroscopic defects in cuprous oxide single crystals grown by the floating zone method in an effort to obtain bulk defect-free crystals without post-growth annealing. The number and distribution of defects in as-grown single crystals are compared to both the polycrystalline rods prior to crystal growth and crystal slices after annealing. The morphology is also examined because both crystals and their inclusions, owing to anisotropic surface energy, do not grow with perfect circular cross sections.

## 2. Materials and methods

The methods for thermal oxidation of feed rods, floating zone crystal growth, and annealing were previously described [23]. X-ray diffraction results are also described in that paper. Briefly, polycrystalline feed and seed rods of cuprous oxide were obtained through thermal oxidation of copper metal rods. Grooves were machined into one end of the feed rod prior to oxidation. The groove allows for the feed rod to be easily aligned in the floating zone furnace. Metal rods were etched in 1M HCl for about 1 h and rinsed with water. Metal rods were then placed horizontally on supports made from copper metal foil on top of an inverted alumina boat. The foil supports were needed to prevent reactions between the metal rod and the alumina. Metal rods were then oxidized in a furnace at 1045 °C for 3 d. Crystal growth was conducted in an optical image furnace (CSI-FZ-T-10000-H-VI-VP) equipped with four 300 W tungsten halide lamps. Floating zone growth conditions for each sample are shown in table 1. Crystal slices were annealed in a box furnace at 1045 °C for 1–5 d and cooled to room temperature at a rate of 5 °C min^−1^.

**Table 1.** Sample growth conditions. The ∗ indicates variable rotation rate described below.

Sample	Purity (%)	Gas	Growth (mm h^−1^)	Rotation (RPM)
A	99.9	Argon	3.5	7
B	99.99	Air	3.5	7
C	99.99	Air	7.0	7
D	99.9	Air	3.5	∗

## 3. Results and discussion

The basic starting point for oxidation of copper to cuprous oxide is

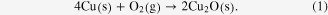
The kinetics of this reaction has been measured [37]. The material can be further oxidized at lower temperatures or higher oxygen pressures to cupric oxide

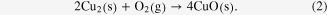
Incomplete equilibration, however, produces some significant complications. The dominant transport mechanism in cuprous oxide is the outward diffusion of copper atoms via copper vacancies [29, 38]. Diffusion of copper radioisotopes [32] and oxygen-18 [39] in cuprous oxide have been measured.

Oxidation of cuprous oxide begins with the formation of a cupric oxide surface, which continues to grow on two fronts [34]. The process starts with the reaction

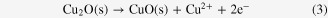
at the Cu_2_O/CuO interface. The Cu^2+^ cations and two electrons migrate through the CuO layer toward the surface to combine with oxygen following the reaction

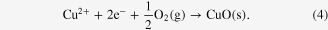


Chemical reactions, however, are not independent of the atomic environment in which they occur.

The slight copper deficiency in Cu_2_O can form through the creation of copper vacancies by

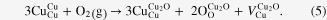
This reaction, written in a modified Kröger–Vink notation of species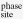 to indicate the atomic environment, does not go to completion in this experiment. Reactions (1) and (5) can be simultaneous. Under more oxidizing conditions, copper vacancies in the cuprous oxide lattice can precipitate as CuO inclusions [23, 26]

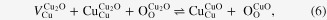
resulting in cupric oxide without direct reactivity with O_2_ in the atmosphere. Similarly, it is possible to form a gaseous void at a cuprous oxide/cupric oxide interface via

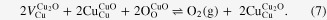
Equations (5)–(7) demonstrate some mechanisms by which vacancies and inclusions can form at low vacancy concentrations.

Equation (7) is not unique for including a gaseous phase, but compared to the alternatives it has the advantage that it results in cuprous oxide which is more stoichiometric. It also does not rely on the existence of any isolated cupric oxide vacancies, which are predicted [40], but whose existence we have not addressed experimentally. This equation is appropriate for explaining the formation of gaseous inclusions in samples formed from a homogeneous melt. For copper oxidized in the solid phase, expansion related stress and grain boundaries are substantial additional factors.

### 3.1. Feed rod preparation

**Figure 1.**
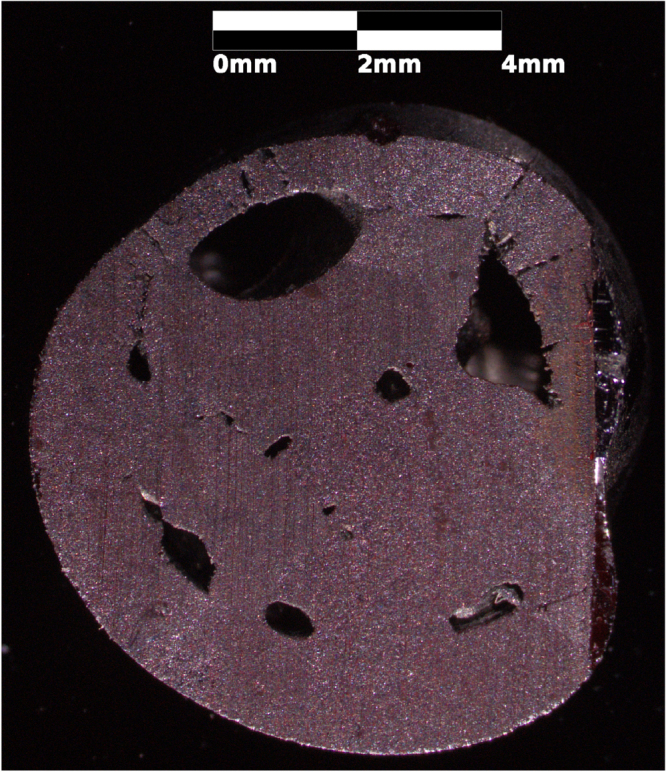
A radial cross-section of a thermally oxidized copper rod. This sample has not been polished. There are multiple black inclusions. Thermally oxidized samples are polycrystalline, though grains are not easily visible without polishing.

**Figure 2.**
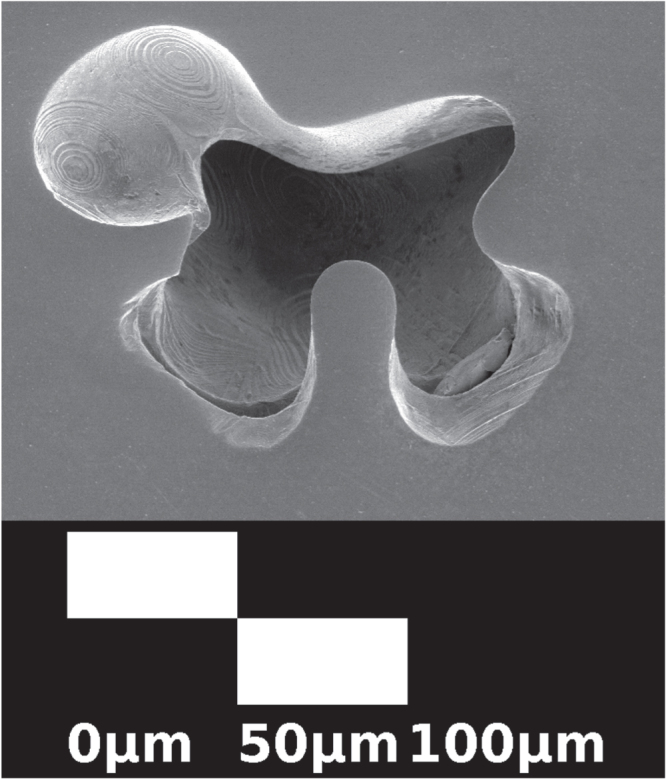
An SEM image of an inclusion in a longitudinally cut and polished slice of the Cu_2_O support rod used to grow sample A. The floating zone grown region is essentially featureless in the SEM. This inclusion is a cavity rather than CuO though it may have a CuO surface layer. There is low contrast between CuO and Cu_2_O in SEM images because the compounds have similar density. Notice the fine faceting on the interior of the inclusion.

The quality of the feed rod can affect floating zone crystal growth: feed rods should be straight and have a uniform density as close to the crystal density as possible [41, 42]. Polycrystalline rods of cuprous oxide were obtained through the thermal oxidation of copper metal rods. While straight and dense rods (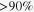 crystal density) were obtained through the thermal oxidation of copper metal, defects were still present. Figures 1 and 2 show defects on the mm and *μ*m scale, respectively. Several voids can be seen in a radial cross-section of the thermally oxidized rod in figure 1. Smaller voids can be seen using scanning electron microscopy (SEM), as shown in figure 2. The image was collected with a MIRAII TESCAN at 7 kV with a secondary electron detector. These defects are significantly reduced during floating zone crystal growth.

**Figure 3.**
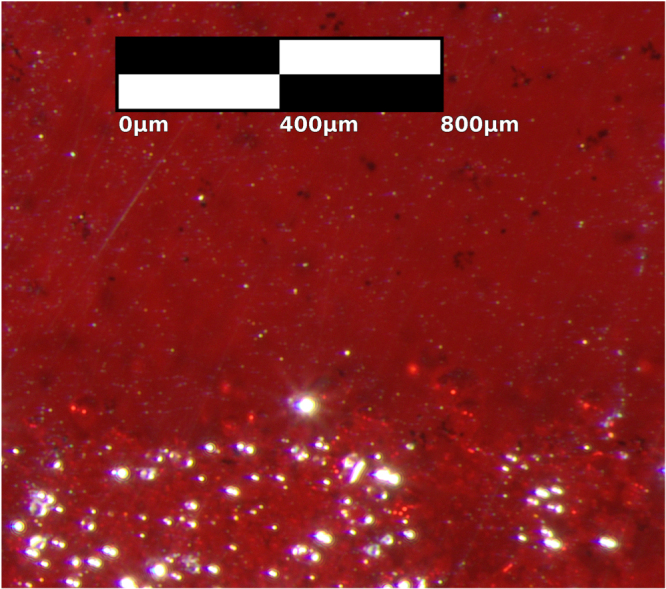
Transmission microscopy image of a polished slice of a cuprous oxide crystal (sample A) that shows the horizontal boundary between the feed rod that was melted (top) and the unmelted support rod (bottom). The boundary is about one third of the way up the image. Dark regions are CuO in the bulk. Light regions are surface defects, which scatter light. The surface defects in the unmelted support rod outnumber all macroscopic defects in the bulk of the melted region. Melting and recrystallization therefore dramatically improves the phase purity of cuprous oxide.

Figure 3 shows an optical microscopy image of a polished slice of a cuprous oxide crystal where the feed and seed rods first connected, cut parallel to the direction of growth. Dark regions are CuO inclusions beneath the crystal surface. Bright regions are defects on the surface, which scatter light. The surface defects alone in the polycrystalline seed rod are more prevalent than all macroscopic defects in the crystallized region. The sample was oxidized in the box furnace but not in the floating zone furnace. In this case the box furnace atmosphere was air but the floating zone furnace atmosphere was argon.

### 3.2. Crystal growth

It is reasonable to think that cupric oxide initially contained in the feed rod causes cupric oxide inclusions to persist in crystals after floating zone growth. Equilibrium may not be achieved in the molten zone in all systems, i.e. not all the cupric oxide is reduced to cuprous oxide. In our experiment, however, results suggest that the oxidation state of copper reaches equilibrium in the melt.

The growth rate was varied to manipulate the time available for the material in the molten zone to reach equilibrium. The growth rate ranged from 3.5 to 17.5 mm h^−1^. If cupric oxide inclusions or liquid were present in the melt (which is around 1125 °C in air [26] and has thermal gradients) the section grown at a faster rate should have a greater number and/or larger cupric oxide inclusions. No qualitatively significant difference was found, however, in the size or number of inclusions for the growth rates examined, which suggests that equilibrium is achieved in the molten zone, and the cupric oxide inclusions form in the solid after crystallization.

For further validation, a single crystal was recrystallized through a second floating zone growth cycle to determine if equilibrium is achieved in the molten zone. A second growth cycle did not have a significant effect on the size and number of cupric oxide inclusions, which confirms that cupric oxide inclusions do not persist in the molten zone and instead form inside the crystal upon cooling.

When crystals are grown in air, a cupric oxide film grows on the crystal surface. When crystals are grown under argon, the cupric oxide surface film does not grow, but inclusions continue to form. Previously we reported the advantages of growing crystals in an oxidizing atmosphere [23]. Varying the oxygen partial pressure changes the cuprous oxide melting point [26]. The fact that cupric oxide inclusions persist in crystals when grown under argon and are not dependent on growth rate or amount of cupric oxide in the feed rod suggests that the inclusions can only be manipulated by changing how the crystal cools. There are two ways to potentially affect the cooling profile of the crystal as growth progresses other than a change in the growth rate: the power of the lamps and the rotation rate of the feed and seed rods.

The dependence of the temperature profile of a crystal in a similar four-mirror floating zone furnace on the operating lamp power has been previously described. While the temperature gradient does initially increase with increasing lamp power, the average increase in temperature per unit length from the hottest point starts to plateau at around 50% lamp power [43]. We have previously demonstrated that 56.8–60.8% lamp power was the approximate power range at which cuprous oxide crystals could be grown. Crystals grown within this temperature range therefore will experience very similar cooling curves. This hypothesis is supported by the fact that the copper vacancy concentration does not vary as a function of lamp power [23]. Since copper vacancy concentration and cupric oxide are related by equation (6), the cupric oxide inclusions should also not be significantly influenced by lamp power.

While the counter-rotation of the seed and feed rods does not ultimately affect the average copper vacancy concentration either [23], faster rotation causes forced convection which decreases thermal gradients. Thermal gradients in and temperature homogeneity of the molten zone can affect how the crystal below the molten zone cools as growth continues and in turn affect the size, number, and radial distribution of inclusions in the crystal.

**Figure 4.**
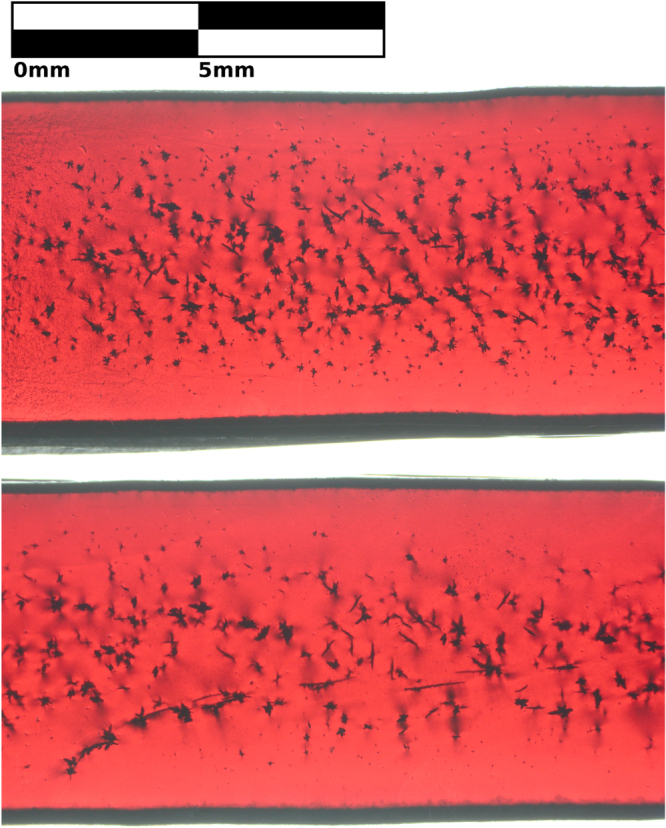
Transmission microscopy image of two sections of sample D that were grown with different counter-rotation rates of feed and support rods. Top: 70 RPM per rod. Bottom: 7 RPM per rod. The counter-rotation rate changes the size, number, and distribution of CuO inclusions, which are small, dark spots.

To clearly demonstrate the effect of rod rotation rate on inclusions, figure 4 shows a longitudinal slice cut parallel to the direction of growth through the approximate center of rod D and compares the size and number of inclusions of samples grown with 7 RPM and 70 RPM rotation rates. Figure 4 was taken through parallel polarizers, so it shows stress induced birefringence near the inclusions and edges.

**Figure 5.**
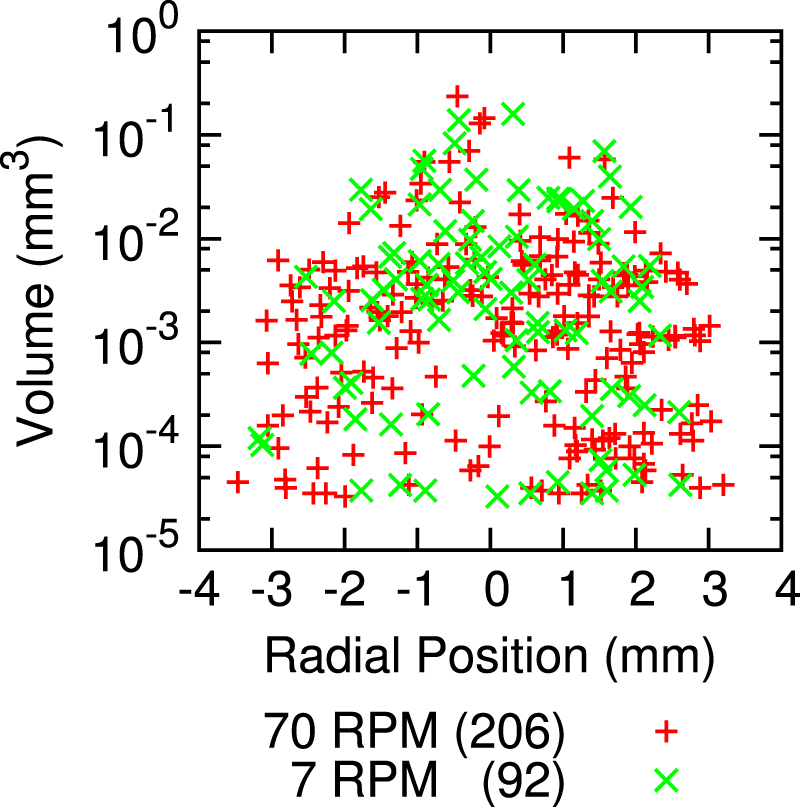
Threshold particle analysis of crystal segments grown with counter-rotation rates of 70 RPM per rod (+ symbol) and 7 RPM per rod (× symbol). The volume and radial position of each inclusion is shown. The inclusions in the crystal grown with a faster rotation rate are more numerous, smaller, and spread farther from the center of the sample. Crystals analyzed were from sample D and are shown in figure 4.

Figure 4 was examined with threshold particle analysis [44]. The quantity, size, and radial distribution of inclusions are summarized in figure 5. There are 2.2 ± 0.3 times as many inclusions in the region grown with a rotation rate of 70 RPM (*p* < 0.000 01, one tailed exact Poisson test [45]). Inclusions are 1.9 ± 0.2 times larger in volume when a rotation rate of 7 RPM is used (*p* < 0.05, two sample t-test [45]). With respect to the total volume fraction of inclusions, the volume increase of individual inclusions does not compensate for the smaller number of inclusions; the volume fraction of inclusion is greater for a fast rotation. In this case, there is a deviation from the suggested inverse relationship between inclusion size and number [27] owing to the exchange between inclusions and vacancies.

Owing to sintering at the surface and slower cooling in the interior, most of the included cupric oxide is near the center of the rod for slow rotation. However, inclusions are spread out 20% further radially when the rotation rate is faster (*p* < 0.01, F-test [45]). Most applications (e.g. [1, 3]) benefit from using a slow rotation rate to minimize the volume fraction of the inclusions and make the inclusions more concentrated so they are easily avoided.

### 3.3. Annealing

We have previously shown how phase separation of cupric oxide from cuprous oxide can be achieved if samples about 1 mm thick are annealed in air from 1045 °C and cooled at 5 °C per minute. Annealing eliminates CuO inclusions via the reverse of equation (6). Gradual cooling rate allows equation (6) to operate close to equilibrium, where the copper vacancies diffuse to the location where CuO will have the lowest energy before undergoing the forward direction in equation (6). The end result is that most CuO sinters to the surface, where it can easily be removed through the polishing process.

**Figure 6.**
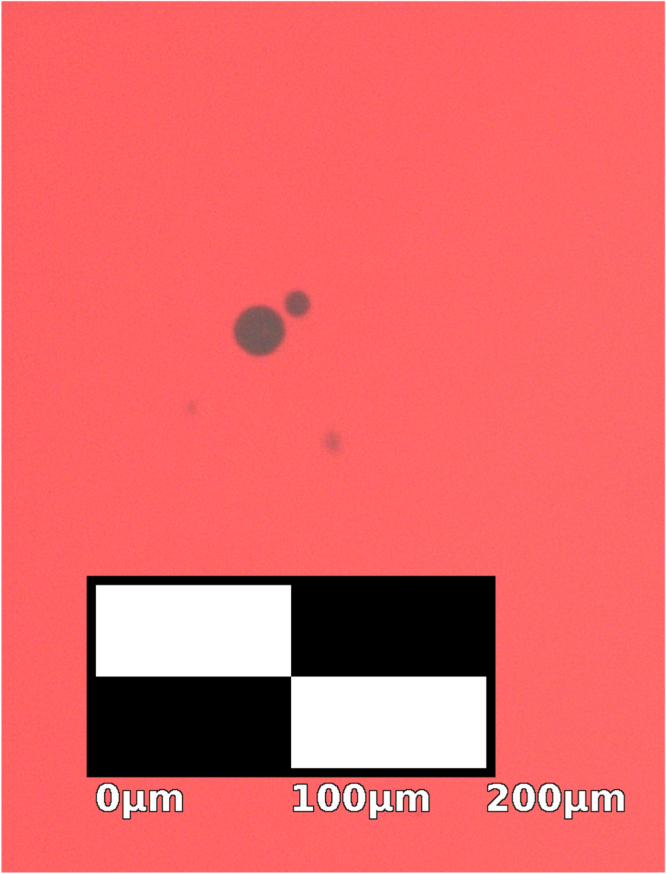
A transmission microscopy image of black CuO inclusions in a Cu_2_O crystal slice from sample B that has been annealed for 5 d at 1045 °C with heating and cooling rates of 5 °C min^−1^. The annealed inclusions are small and rounded, but show faceting in contrast with the inclusions before annealing in figure 4.

While most cupric oxide inclusions can be removed with this process, some inclusions do persist in annealed samples. Inclusions in oxidized and floating zone crystallized samples that have not been annealed are highly irregular in shape and quite large [26, 27]. Figure 6 shows residual inclusions under an optical microscope after annealing, which are much smaller and rounded with a slight Wulff-construction-like faceting owing to anisotropic surface energy. The bulk crystal is also subject to anisotropic surface energy during growth [27, 46–48]. Phenomenologically, the observed shape of sample B, which was grown in the 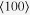 direction, is a squircle [49, 50]. A squircle with radius *r* is described by

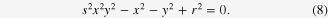
The observed squareness *s* = 0.564.

Threshold particle analysis was performed on crystal slices with and without a post growth annealing (see figures 8(a) and (d) in [23]). The inclusion volume fraction in the interior improved from 0.28 ± 0.02 to 0.00051 ± 0.00007, which is a factor of 540 ± 70 improvement in this particular sample. While we have previously demonstrated how CuO quantities in annealed samples are too small to measure with powder x-ray diffraction, they can be measured very accurately using a transmission microscope. The inclusion fraction in annealed samples does vary. It is quite costly to collect statistics on this variation because the volume fraction is so low, but in all cases observed the inclusion fraction is greatly improved after annealing.

### 3.4. Grain boundaries

Though previous studies reported grain nucleation in cuprous oxide grown directionally from a melt [24, 25], in 35 trials no grain nucleation was observed. The improved reliability of growth may be explained by our use of a four lamp furnace design [43], which provides more stable heating and cooling. However, if a polycrystalline support is used to grow the crystal, occasionally grain boundaries may persist in the sample until they are eliminated through random walk or differences in the growth rate in different crystallographic directions. Frequently the remaining grain is growing in a direction close to [100].

**Figure 7.**
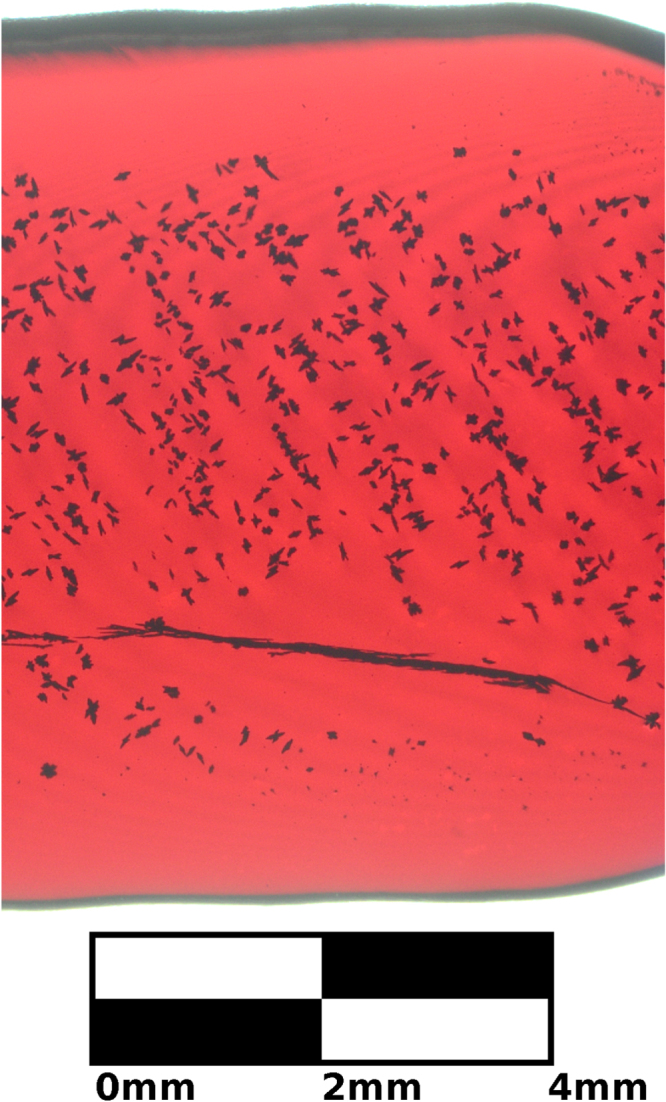
A transmission microscopy image of sample C that shows black CuO inclusions accumulate along a grain boundary. The area around the grain boundary is free of inclusions. Grain boundaries can be avoided if crystals are grown from a single crystal seed rod.

An example of a grain boundary in a polished slice under an optical microscope is shown as a dark, uneven horizontal line in figure 7. The boundary is marked by an accumulation of inclusion material along the two-dimensional grain surface, which is viewed nearly edge on. The surrounding area is depleted of inclusions. This indicates that there is an attractive minimum in the chemical potential of defects near grain boundaries, which would trap vacancies and prevent CuO from precipitating on the surface of the crystal. The energy of a grain boundary plus the energy of an inclusion in different locations is greater than the energy of both in the same location. Therefore preparation of a single crystal improves transparency.

## 4. Conclusions

Floating zone growth followed by annealing is an excellent means of obtaining cuprous oxide crystals for optical applications. Transport of copper vacancies through the cuprous oxide lattice, however, leads to the formation of cupric oxide and gaseous inclusions. Proper annealing procedures eliminate inclusions in thinner samples using vacancy transport and surface energy minimization to separate phases. For applications where thicker samples are needed, however, it is difficult to obtain a larger, thicker crystal without cupric oxide inclusions.

Cupric oxide inclusions persist in floating zone crystals, even if a cuprous oxide single crystal is used as the feed rod. The rotation rate was the only variable that had a qualitative effect on the size and distribution of inclusions. While inclusions are always present after crystal growth, a slower rotation rate provided a smaller number and volume fraction of inclusions at the expense of a larger average inclusion size. Larger inclusion size can make inclusions easier to locate and avoid. The phase and structure of small inclusions are influenced by minimization of interfacial energy between phases and grains, as is the overall crystal morphology. Optical microscopy effectively characterizes phase purity, morphology, and grains, which has allowed us to demonstrate the conversion of opaque cuprous oxide to transparent crystals.
